# Soybean Oil, Linoleic Acid Source, in Lamb Diets: Intake, Digestibility, Performance, Ingestive Behaviour, and Blood Metabolites

**DOI:** 10.3390/ani14142075

**Published:** 2024-07-16

**Authors:** Victor G. O. Lima, Liliane O. da Silva, José E. de Freitas Júnior, Henry D. R. Alba, Willian P. Silva, Douglas dos S. Pina, Laudí C. Leite, Carlindo S. Rodrigues, Stefanie A. Santos, Carly A. Becker, Gleidson G. P. de Carvalho

**Affiliations:** 1 Department of Animal Science, Universidade Federal da Bahia, Av. Adhemar de Barros, 500, Ondina, Salvador 40170110, Brazil; victorgolima@hotmail.com (V.G.O.L.); lillioliveira9@gmail.com (L.O.d.S.); jose.esler@ufba.br (J.E.d.F.J.); harrydoo@gmail.com (H.D.R.A.); willianzoo@yahoo.com.br (W.P.S.); douglas.pina@ufba.br (D.d.S.P.); carlindo.rodrigues@gmail.com (C.S.R.); stefanie.alvarenga@ufba.br (S.A.S.); 2 Department of Animal Science, Universidade Federal do Recôncavo da Bahia, Cruz das Almas 44380000, Brazil; laudi.ufrb@gmail.com; 3 Penn State Extension, College of Agricultural Science, Pen State University, State College, PA 16802, USA; cab7033@psu.edu

**Keywords:** feedlot, feeding efficiency, lipids, ruminant nutrition, sheep, supplemental oil

## Abstract

**Simple Summary:**

Enhancing the energy density of lamb diets is a proven method to improve their productivity. While using carbohydrates for this purpose may seem intuitive, it can lead to undesirable shifts in ruminal metabolism, potentially triggering metabolic diseases. Hence, substituting carbohydrates with lipids is favored. Nevertheless, lipids, though effective, can also disrupt ruminal fermentation parameters. Among these lipids, soybean oil stands out for its positive impact on both beef and dairy cattle, leading to improved performance. In our study, we sought to assess the effects of soybean oil supplementation on performance, digestibility, feeding behaviour, and blood metabolites of feedlot lambs. Our findings indicate that the inclusion of soybean oil resulted in a linear reduction in nutrient intake, consequently lowering the average daily gain of the lambs. Additionally, soybean oil supplementation induced selective feeding behaviour among the lambs. Based on our observations, we recommend incorporating soybean oil into lamb diets at a maximum inclusion rate of 41 g/kg DM to optimize production parameters. The strategy implemented to adapt lambs to increasing levels of high-fat diet mitigated the detrimental effects of lipids on the rumen, with high-density energy intake being the constraining factor on performance.

**Abstract:**

The objective of the current study was to evaluate the effects of soybean oil inclusion in diets on feeding behaviour, digestibility, performance, and blood metabolites of feedlot lambs. Forty non-castrated Santa Inês lambs with a mean age of 5 months and initial body weight of 34.88 ± 3.19 kg were used in a 40-day feeding trial. The lambs were distributed in five experimental diets with the inclusion of increasing soybean oil (SO) levels: 0, 30, 60, 90, and 120 g/kg DM. The SO inclusion promoted a linear reduction in DM intake (*p* < 0.001), crude protein (CP, *p* < 0.001), non-fibrous carbohydrates (NFC, *p* < 0.001), and total digestible nutrients (TDN, *p* = 0.004). There was an increasing quadratic effect on the intake of ether extract (EE; *p* = 0.002) and decreasing for neutral detergent fiber (*p* = 0.005). The soybean oil inclusion promoted the greater apparent digestibility of CP (*p* = 0.016), EE (*p* = 0.005), NDFom (*p* < 0.001), and TDN (*p* < 0.001); on the other hand, the apparent digestibility of NFC (*p* = 0.005) was decreased. The average daily gain decreased (*p* < 0.001) with SO inclusion. The SO inclusion increased feeding time (*p* = 0.004), reduced the efficiency of DM rumination (*p* = 0.001), and reduced the concentration of blood N-ureic (*p* < 0.001). Considering the productive parameters, SO can be included in diets and it is recommended that we include SO of up to 41 g/kg DM in diets for fattening lambs as the ideal maximum level. The strategy implemented to adapt lambs to increasing levels of high-fat diet mitigated the detrimental effects of lipids on the rumen, with high-density energy intake being the constraining factor on performance.

## 1. Introduction

In recent decades, human nutrition has been based on a higher intake of readily available carbohydrates, processed foods, saturated fats, and salt. However, in recent years, the population’s lifestyle and dietary habits have changed due to increased knowledge about the effects of the different molecules present in food [1]. Therefore, there is a greater emphasis for green and healthy functional foods.

Linoleic acid (LA) and its isomers, such as conjugated linoleic acids (CLAs), are functional and essential fatty acids. These metabolites are essential in human nutrition due to their participation in various metabolic pathways, acting as precursors to essential metabolites and contributing to improved health status [2]. Oilseeds, nuts, legumes, eggs, meats, and dairy products are sources of LA. Increased LA sources in ruminant diets results in a higher LA and CLA content in ruminant products [3,4,5,6]. Linoleic acid is the fatty acid (FA) with the highest concentration in soybean oil, containing 52.23 g/100 g of total fatty acids [4] or 52.91–54.79 g/100 g of total FA [6,7,8].

However, the use of lipids in ruminant diets is very important in animals with a decreased feed intake. Nevertheless, their use is controversial because, despite increasing the energy density of the diet, at the same time, its use is correlated with a certain degree of toxicity for rumen microbiology [9,10]. Several reports indicate that the inclusion of LA sources does not compromise dry matter intake (DMI), fiber digestibility [7,11], or lamb performance [5,12]. At the ruminal level, the inclusion of soybean oil in approximately 7 g of ether extract/kg DM for growing lambs promoted a greater development of papillary width (33.6% greater), thickness of the epithelium (57.1% greater), thickness of the submucosal layer (60.9% greater), and thickness of the muscle layer (50.1% greater) compared to a diet without the inclusion of soybean oil [13]. Furthermore, it is important to note that the inclusion of LA sources in lamb diets can increase feeding efficiency [12,14], increase unsaturated and polyunsaturated fatty acid (UFA) contents in meat [15], and reduce enteric methane production in lambs [16]. 

However, undesirable effects have also been reported from the addition of LA sources in lamb diets. A reduction in the voluntary intake of dry matter (DM) and the digestibility of nutrients, specifically fiber, was observed [14,15,17]. In the rumen, one of the primary mechanisms employed by bacteria to mitigate the adverse effects of PUFAs is biohydrogenation (BH). This process operates at an approximate rate of 89% for LA, resulting in the production of vaccenic acid (18:1 trans-11), which can partially inhibit BH, thereby increasing the flux of PUFAs to the intestine [7,18,19]. PUFAs are absorbed and delivered to the liver, where they are oxidized to produce metabolizable energy that animals use to achieve maximum productive performance [20]. The negative effects of soybean oil supplementation in diets can be minimized with a proper adaptation of the animals, because this can lead to a concomitant adaptation of the rumen microbes and the physiological functions of the rumen epithelium to this type of diets [13].

In this sense, it was hypothesized that there is an ideal level for the inclusion of lipids, assuming that higher levels of soybean oil can be included in the diet of fattening lambs. The objective of the present study was to evaluate the inclusion of up to 120 g of soybean oil in the DM basis of the diet on the feed efficiency, productive performance, and blood metabolites of feedlot lambs. Lipid supplementation has been extensively documented in the literature. Nevertheless, controversial data suggest that its influence on ruminant feeding, metabolism, and performance is a topic of concern. Studies of this nature are crucial for elucidating significant factors that can guide farmers in their quest for optimal alternatives in ruminant production, especially under tropical conditions characterized by limited feed availability during certain seasons and the impact of climate on feed intake.

## 2. Materials and Methods

### 2.1. Location and Ethical Considerations

The experiment was carried out at the Experimental Farm of the Veterinary Medicine and Animal Science School—Universidade Federal da Bahia, located in the municipality of São Gonçalo dos Campos, Bahia, Brazil, at 12°23′57.51″ S and 38°52′44.66″ W. The experimental procedures were carried out with the authorization of the Ethics Committee in the Use of Animals of the Universidade Federal da Bahia, under protocol No. 72/2018. The regional climate, according to Wilhelm Köppen’s climate classification, is type As—“A” tropical climate with average monthly temperatures above 18 °C and “s” indicating a dry season during the period of greatest sunshine and longest days.

### 2.2. Animals, Design, and Experimental Diets

Forty non-castrated male Santa Inês lambs were used, with a mean age of 5 months and an initial body weight (IBW) of 34.88 ± 3.19 kg (mean ± SD). The lambs were housed in 1.2 m^2^ individual pens, covered, with a wooden slatted floor, and suspended; and provided with drinkers and feeders with unrestricted access to water and experimental diets. At the beginning of the experiment, lambs were identified, controlled for endoparasites and ectoparasites, immunized with a polyvalent vaccine against clostridia, and supplemented with vitamins A, D, and E.

The total duration of the experiment was 55 days, where 15 were dedicated to the adaptation of lambs to the environment and diets (adaptation period) and 40 days to data collection (experimental period). During the adaptation period, SO was gradually incorporated into the diets. Initially, 10 g of SO/kg DM was incorporated into the diets, increasing daily until reaching the levels used in the experimental diets. At the beginning of the experimental period, lambs were weighed after a 16 h fast, obtaining the initial body weight. The diets were provided in two meals a day, 50% at 8:00 am and 50% at 4:00 pm. Feed was supplied to allow 10 to 20% of refusals. The experimental diets consisted of sorghum (*Sorghum bicolor*) silage as a forage source, with an average particle size of 5 cm, and concentrate, consisting of soybean meal, ground corn, soybean oil, urea, and a specific commercial mineral supplement for sheep. It is important to note that the inclusion of SO partially replaced corn grain and soybean meal (Table 1).

The experimental diets were composed of five levels of SO inclusion: 0, 30, 60, 90, and 120 g/kg DM (Table 1). Lambs were distributed within the treatments following a completely randomized design. Diets were formulated to be isoproteic and similar in energy content following the recommendations of the National Research Council [9] to meet the requirements for lambs with an estimated average daily gain (ADG) of 200 g/day. Diets were prepared daily to prevent the rancidity of the SO.

### 2.3. Ingestive Behaviour

On days 12 and 32 of the experimental period, all lambs were subjected to visual observation to evaluate the ingestive behaviour. Eight trained observers, divided into 2 groups, took turns conducting observations every 5 min for 24 h to determine the time spent on feeding, ruminating, and idling activities [21]. On the same day, the number of rumination jaw movements and the time spent ruminating the ruminal boluses were counted, using a digital stopwatch. For this evaluation, observations of three ruminal boluses were made, in three different periods of the day (1000 to 1200, 1400 to 1600, and 1900 to 2100 h), measuring the average number of rumination jaw movements and the time spent per ruminal bolus. During the night, the environment was kept under artificial lighting, with previous adaptation before the start of the experiment.

Feeding (FEE) and ruminating (RUE) efficiencies, total chewing time per day (TCT), number of ruminated boluses per day (NB), number of ruminations per boluses (NR), and rumination time (RT) were obtained according to the methodology described by Polli et al. [22] and Bürger et al. [23].

### 2.4. Nutrient Intake, Digestibility, and Performance

Refusals were collected and weighed daily before the first feed supply using a digital scale (SHI-UX-6200H, TECNAL, São Paulo, Brazil) to obtain the dry matter intake (DMI). 

The digestibility trial was performed in two periods of five days each, on days 14 to 18 and days 35 to 39 of the experimental period. Faeces were collected directly from the rectum of the lambs twice (morning and afternoon), at intervals of 8 h. Samples were placed in identified plastic bags and stored at −20 °C. Subsequently, samples were pre-dried, ground to 2 mm (50%) and 1 mm (50%), and pooled for further analysis. To estimate faecal production, samples of refusals, faeces, and ingredients were analyzed to determine the content of indigestible NDF (NDFi) using non-woven (TNT) bags in an in situ incubation procedure for 288 h [24].

The digestibility coefficient (DC) of the nutritional fractions (DM, ash, CP, EE, aNDFomp, and NFC) was determined by the following formula:

DC = [(Nutrient consumed (g) − Nutrient in faeces (g))/Nutrient consumed (g)] × 100.

where g of nutrient consumed = g of nutrient supplied − g of nutrient in refusals.

The total weight gain (TWG) was obtained by the difference between IBW (day 1) and final body weight (FBW; day 40). The average daily gain (ADG) was calculated by equation, TWG/40 days. Lambs were weighed in the morning after a solid-fasting period of 16 h. The feed efficiency (FE) was determined through the relationship between the average daily gain (ADG) and the DMI.

### 2.5. Laboratory Analyses 

Samples of ingredients, diets, and refusals were collected and stored in a −20 °C freezer. The laboratory analyses were carried out in the Animal Nutrition Laboratory (LANA) of the Faculty of Veterinary Medicine and Animal Science—UFBA. After thawing at room temperature, samples were pre-dried in a forced air circulation oven at 55 °C for 72 h. Immediately, samples were ground in a Willey type mill (Tecnal, Piracicaba, São Paulo) with a 1 mm sieve to determine the content of DM (method 934.01), ash (method 930.05), crude protein (CP; method 981.10), and ether extract (EE; method 920.39), according to the official methods of analysis of AOAC International [25]. The contents of neutral detergent fiber (NDF) and acid detergent fiber (ADF) assayed with a heat-stable amylase and expressed exclusive of residual ash and protein (aNDFomp and ADFomp) were determined according to Mertens [26] and Licitra et al. [27]. Lignin was determined by treating ADF residues with a 72% sulfuric acid solution. The content of neutral detergent (NDIP) and acid detergent (ADIP) insoluble protein was determined according to Licitra et al. [27].

The non-fibrous carbohydrate (NFC) content of the diets were calculated according to Hall [28]. Total digestible nutrient (TDN) levels were estimated using the equations for small ruminants proposed by Cruz et al. [29]. Digestible (DE) and metabolizable (ME) energy were estimated according to the NRC [30] equations.

Ingredient samples for fatty acid profile analysis were lyophilized and uniformly ground (Coffee grinder, Cadence, Santa Catarina, Brazil). The extraction and methylation of samples were carried out by direct synthesis of fatty acid methyl esters (FAMEs) [31]. The fatty acid composition of FAME was identified and quantified using a gas chromatograph (CGFOCUS with split injection) equipped with an SPTM-2560 capillary column (100 m × 0.25 mm ID with 0.02 µm film thickness; Supelco) and flame ionization detector (Thermo Scientific Inc., São Paulo, Brazil). The detector and injector temperatures were set at 250 °C, with a 15:1 split ratio.

The initial oven temperature was maintained at 70 °C for 4 min, subsequently increased by 13 °C/min at 175 °C, maintained for 27 min, and subsequently increased by 4 °C/min at 215 °C, and then maintained for 31 min [32]. Fatty acids were identified by comparing their retention times with the internal standard C 19:0 (189-19 Sigma Aldrich; 10 mg C19:0/mL MeOH).

### 2.6. Blood Parameters

On day 40 of the experimental period, 10 mL of blood was collected in vacutainer tubes, with coagulant and anticoagulant to obtain the serum and plasma, respectively, four hours after the first feeding through jugular vein puncture. Samples were centrifuged at 1500 rpm× *g* for 15 min. Immediately, the serum and plasma were transferred to Eppendorf microtubes and stored at −20 °C for later analysis.

The serum concentrations (mg/dL) of total protein and albumin were carried out using commercial kits (Labtest^®^, Minas Gerais, Brazil) and a spectrophotometer (wavelengths of 550 nm and 630 nm, respectively). The globulin content was calculated as the difference between total proteins and albumin. The albumin:globulin ratio was obtained by dividing albumin and globulin fractions.

Serum levels of N-urea, triglycerides, and plasma glucose were determined using commercial kits (Doles^®^, Goiás, Brazil). Total cholesterol was determined using a commercial kit (Labtest^®^, Minas Gerais, Brazil). A spectrophotometer was used to measure the content of urea and triglycerides (600 nm), glucose (510 nm), and total cholesterol (500 nm).

The activity of the enzymes, alanine aminotransferase (ALT), aspartate aminotransferase (AST), and gamma-glutamyltransferase (GGT) were measured using commercial kits (doles^®^, Goiás, Brazil) and a spectrophotometer (SBA 200^®^, CELM, São Caetano do Sul, Brazil).

### 2.7. Statistical Analyses 

The experimental design was completely randomized with five diets containing SO, 0, 30, 60, 90, and 120 g/kg DM, and eight replicates (lambs). The PROC MIXED of SAS (Statistical Analysis System, version 9.4) was used. The treatments were randomly assigned to experimental units using the PROC SURVEYSELECT procedure of SAS system. Normality was assessed using the Shapiro–Wilk test and assumptions of homogeneity of variance were assessed using the Bartlett test. The results were subjected to analysis of variance, and the effects of the inclusion SO were studied using polynomials, linear, and quadratic contrasts. The 0.05 level was determined as the critical probability level. The regression models were selected based on the coefficients of determination and the significance of the regression coefficients and was used to determine the ideal level of SO inclusion considering the ADG of lambs. Initial body weight was used as a covariate for performance variables analysis. The data were analyzed using the model:

$$Y_{ij}=\mu +\alpha_{i}+\beta (X_{ij}-X)+{Ɛ}_{ij}$$

where Y_ij_ = observed value of the dependent variable; µ = overall mean; α_i_ = effect of treatment i (i = 1 to 5); β = common slope parameter; X_ij_ = initial body weight of animal j on treatment i (covariate); X = mean value for covariate; and Ɛ_ij_ = residual error.

## 3. Results

### 3.1. Nutrient Intake, Digestibility, and Performance

Increasing levels of soybean oil (SO) inclusion promoted a linear reduction in the intake of DM (*p* < 0.001), CP (*p* < 0.001), NFC (*p* < 0.001), and TDN (*p* = 0.004) (Table 2).

A quadratic effect (*p* = 0.002) on EE intake with a maximum intake (173.01 g) at the SO inclusion level of 145.99 g/kg DM occurred. On the other hand, the lowest intake (*p* = 0.005) of aNDFomp (190.56 g) was estimated at the SO inclusion level of 111.26 g/kg DM. Furthermore, there was a quadratic effect (*p* = 0.010) on aNDFomp intake (g/kg BW), with a minimum intake (5.1 g) at the inclusion level of 87.5 g/kg DM.

The apparent digestibility of CP (*p* = 0.016), aNDFomp (*p* = 0.033), and EE (*p* = 0.005) and the TDN content (*p* < 0.001) increased linearly with the SO inclusion in diets. The apparent digestibility of NFCp (*p* = 0.005) decreased linearly (Table 2).

A linear reduction was observed for FBW (*p* < 0.001) and ADG (*p* < 0.001). However, the feeding efficiency (*p* = 0.026) was quadratic (Table 2), with a maximum efficiency (188.27 g BW gain/kg DMI) at the SO inclusion level of 27.19 g/kg.

### 3.2. Ingestive Behaviour

There was an increase in feeding time (hours/day; *p* = 0.004). The rumination and idling times were not affected (*p* > 0.05). The feeding and rumination efficiencies of DM and NDF decreased (*p* < 0.05) as a function of the SO inclusion level (Table 3).

The variables chews g DM/bolus (*p* = 0.037), NR (*p* = 0.003), and RT (*p* = 0.003) showed a quadratic behaviour with maximum observations at SO inclusion levels of 60, 65.63, and 68.01 g/kg DM in diets. Regarding NB, which was also quadratic (*p* = 0.040), a minimum value (468.23 ruminal boluses) was observed at the SO inclusion level of 81.11 g/kg DM. Total chewing time (TCT) was not affected by the inclusion of SO.

### 3.3. Serum Metabolites

There was an increase in the concentration of total protein (*p* < 0.001) and globulin (*p* < 0.001) with the SO inclusion at all SO inclusion levels (Table 4). The albumin:globulin ratio (*p* < 0.001) was negatively affected by the SO inclusion. Blood urea N concentrations were reduced (*p* < 0.001).

Regarding the energy profile, the glucose concentration showed a decreasing linear behaviour (*p* < 0.001) with SO inclusion. However, the triglyceride concentration showed a quadratic behaviour (*p* < 0.001), in which the lowest value (27.38 mg/dL) was observed at the SO inclusion level of 30 g/kg DM. Total cholesterol concentrations were not affected (*p* = 0.910).

The liver enzyme activity was affected with the SO inclusion in diets, resulting in higher AST concentrations (*p* < 0.001). There were no differences in the activity of ALT (*p* = 0.287) and GGT (*p* = 0.058) enzymes (Table 4).

## 4. Discussion

### 4.1. Nutrient Intake and Performance

The reduction in DMI by lambs is consistent with the liver oxidation theory [33]. The long-chain fatty acids (LCFAs) present in SO are metabolic fuels oxidized in the liver to increase ATP production, promoting the suppression of DMI [20,34]. In the current study, LCFA increased as SO was included (Table 1), mainly unsaturated fatty acids (UFAs), oleic acid, and linoleic acid contents. Kucuk et al. [35] reported that elevated SO levels increased the duodenal flux of fatty acids in lambs. Additionally, high-UFA diets can increase the production of cholecystokinin in the small intestine. This enzymatic hormone increases the retention time in the rumen and reduces rumen emptying, promoting rumen distension and satiety, consequently decreasing feed intake [36]. Furthermore, considering the notable increase in UFA content, a decrease in DM was expected, as UFAs can be toxic to ruminal bacteria, mainly fibrolytic bacteria, as observed by other researchers [3,14,15,17,18]. The decrease in DMI resulted in a lower intake of other nutritional components of the diet, except EE. The SO inclusion increased the EE content of the diet, explaining the higher EE intake with the SO inclusion of 120 g/kg DM. DM digestibility in the current experiment was not affected by SO inclusion in the diet. On the other hand, NDF digestibility increases due to the reduction in NDF intake (Table 2). This observation, along with the increased EE digestibility, indicates that EE was not a limiting factor in ruminal digestion. This is likely due to the higher biohydrogenation rate and/or passage rate of the nutritional components to the small intestine [7,19]. This would explain the EE digestibility, considering the high LCFA digestibility in the small intestine [4]. In ruminants, the NFC digestibility is higher in the rumen environment [37]. Therefore, NFC was probably affected by the adhesion capacity and physical barrier formation promoted by SO inclusion [38], which increased the passage rate of NFC, further reducing its digestibility. Based on previous arguments, it can be inferred that the SO oil was absorbed by the concentrate particles, thereby diminishing its impact on ruminal bacteria and NDF digestibility. Additionally, due to the higher lipid content of the concentrate particles, their degradability in the rumen was decreased, leading to reduced NFC digestibility, which also persists in the intestine. However, upon reaching the intestine, lipid molecules from the concentrate particles were absorbed, benefitting from their enhanced digestibility in this location. Hence, it is concluded that the diet adaptation protocol effectively mitigated the adverse effects of lipids on ruminal microbiology, and the decrease in CMS is closely associated with achieving the required energy levels.

Although the inclusion of SO in the lamb diets promoted the increased digestibility of CP, EE, and TDN, the decrease in nutrient intake did not satisfy the nutritional needs of the animals. Consequently, FBW and ADG were affected, decreasing linearly with the inclusion of SO in the diet. The minimum CP and TDN intake required for moderately growing lambs over 30 kg BW is 167 and 790 g/d, respectively [9]. Therefore, the DMI reduction compromised the formulated weight gain for lambs (Table 2) at inclusion levels higher than 30 g/kg of soybean oil.

Therefore, the feed efficiency with the SO inclusion of up to 30 g/kg DM is explained by DM intake reduction and non-differences in lamb weight gain. Similar feed efficiencies were observed in studies that include fat sources in diets to finishing lambs [12,14,37]. SO inclusion up to certain levels (30 g/kg DM) can provide the greater sustainability of lamb fattening systems, which is explained by the greater efficiency of nutrient utilization.

### 4.2. Ingestive Behaviour

The dry matter intake in feedlot lambs fed SO is influenced by the number and size of daily meals. Although the SO inclusion promoted an increase in feeding time, there was a reduction in meal size demonstrated by the decrease in feeding efficiency (Table 3). The values of feeding efficiency values strongly indicate satiety in lambs on high-fat diets [39]. Furthermore, another effect observed with the inclusion of unprotected fat sources in lamb diets is diet selectivity and/or fat toxicity to the ruminal microbiota [18].

The ruminating efficiency of DM and NDF with SO inclusion was influenced by the DMI behaviour, as the rumination time was not altered. A reduction in NB with the SO inclusion of 60 g/kg DM, associated with a decrease in DMI (Table 3), provided a non-theoretical increase in DM per ruminated bolus, resulting in a higher number of bites and chewing time per bolus [13]. Lima et al. [16] found increases in the NRJM/bolus and TRJB in feedlot lambs fed 50 g/kg DM of soybean oil.

### 4.3. Serum Metabolites

Increased total protein concentrations are related to an increase in globulin concentration. This increase in globulin concentration can be explained by the enhancement in CP digestibility and increased EE intake (Table 2), which promotes the greater utilization of fatty acids as metabolic fuels. Although an increase in globulin concentration was observed, resulting in a reduction in the albumin:globulin ratio, the maximum concentrations of 4.62 g/dL falls within the normal range for the species, which is 3.5 to 5.7 g/dL [40].

The increased dietary fatty acid intake of the lambs likely led to a reduction in the availability of intermediate sources for gluconeogenesis and nitrogen, resulting in decreased serum glucose and N-urea concentrations. This behaviour is similar to that observed by other researchers in lamb trials [39,41].

Possibly, up to the 30 g/kg of SO inclusion level, fatty acids were effectively utilized in metabolism to meet growth requirements. However, SO inclusion levels higher than 30 g/kg of DM probably likely caused an imbalance in CP and energy intake. The excess energy from the FA intake is directed towards triglyceride formation. 

Regarding liver function, there were no changes in ALT and GGT concentrations, indicating no signs of liver damage. The activity of the AST enzyme exhibited a linear increase, suggesting a heightened liver metabolism [42]. The increased transaminase (AST) activity can be attributed to a compensatory physiological mechanism involving N recycling to counterbalance the reduction in CP intake [43]. Although there was an increase in AST concentrations, the values remain within the normal range for lambs (60 to 280 IU/L), indicating no liver damage with SO inclusion [13].

## 5. Conclusions

Soybean oil can be included at up to 41 g/kg DM in feedlot lamb diets containing 600 g/kg DM to achieve an average daily gain of 200 g. The inclusion of 60 g/kg DM of soybean oil may be indicated in situations where the cost of traditional ingredients is higher than that of soybean oil, as this level of inclusion provides a weight gain of 189.3 g, close to the recommended 200 g/day. The strategy implemented to adapt lambs to increasing levels of high-fat diet mitigated the detrimental effects of lipids on the rumen, with high-density energy intake being the constraining factor on performance. One of the primary constraints observed in this study was the necessity to prepare diets daily to prevent soybean oil from becoming rancid. However, this limitation may be less significant for breeders managing a small number of animals or when applied to elite livestock.

## Figures and Tables

**Table 1:** Ingredients proportion and chemical composition of soybean oil and experimental diets.

Item	Soybean Oil Level, g/kg Diet DM					Soybean Oil
	0	30	60	90	120	
Ingredients, g/kg DM basis						
Sorghum silage	400	400	400	400	400	-
Soybean meal	116	120	124	128	133	-
Ground corn	461	427	393	359	324	-
Soybean oil	0	30	60	90	120	-
Urea	8	8	8	8	8	-
Mineral supplement ^1^	15	15	15	15	15	-
Chemical composition, g/kg DM basis						
Dry matter, g/kg as-fed basis	663.3	667.8	672.4	677.0	681.7	999.9
Ash	41.6	41.5	41.3	41.1	41.0	-
Crude protein	148.9	147.9	146.8	145.7	145.0	-
Ether extract	30.9	59.6	88.2	116.9	145.5	999.9
aNDFomp ^2^	273.9	270.7	267.5	264.3	261.1	-
Acid detergent fiber	131.2	130.7	130.1	129.6	129.0	-
Hemicellulose	142.7	140.0	137.4	134.7	132.0	-
Cellulose	111.3	110.9	110.5	110.1	109.7	-
Lignin	19.9	19.8	19.6	19.5	19.4	-
Indigestible neutral detergent fiber	75.3	75.0	74.6	74.3	73.9	-
Non-fibrous carbohydrates	519.1	494.9	470.7	446.6	421.9	-
Total digestible nutrients	754.7	774.7	794.6	814.6	834.5	1969.6
Metabolizable energy, Mcal/kg	3.0	3.2	3.4	3.5	3.7	7.7
NDIP, g/kg CP	80.0	78.8	77.5	76.2	74.8	-
ADIP, g/kg CP	18.6	18.4	18.3	18.1	17.8	-
Fatty acid profile, g/100 g total fatty acids						
Caprylic (C8:0)	0.01	0.01	0.01	0.01	0.01	0.01
Capric (C10:0)	0.05	0.05	0.05	0.04	0.04	0.06
Lauric (C12:0)	0.08	0.08	0.07	0.07	0.07	0.09
Miristic (C14:0)	0.56	0.55	0.54	0.53	0.52	0.47
Palmitic (C16:0)	4.67	4.88	5.09	5.29	5.50	14.25
Palmitoleic (C16:1)	0.12	0.12	0.12	0.12	0.13	0.20
Estearic (C18:0)	1.15	1.24	1.34	1.43	1.53	4.52
Oleic (C18:1 n-9)	7.95	8.17	8.40	8.62	8.83	22.91
Linoleic (C18:2 n-6)	12.7	13.8	14.9	16.0	17.1	59.1
α-linolenic (C18:3 n-3)	0.72	0.91	1.10	1.29	1.48	6.75

^1^ Guarantee levels (per kg of active elements): Calcium—110 g, Phosphorus—87 g, Sulfur—18 g, Copper—590 mg, Cobalt—15 mg, Sodium—147 g, Chromium—20 mg, Iodine—50 mg, Manganese—2 g, Selenium—20 mg, Zinc—3.8 g, Fluorine (max)—870 mg, and Molybdenum—300 mg. ^2^ aNDFomp, Neutral detergent fiber assayed with a heat-stable amylase and expressed exclusive of residual ash and protein.

**Table 2:** Intake, digestibility, and performance of feedlot lambs fed diets containing soybean oil.

Item ^1^	Soybean Oil Level, g/kg Diet DM					SEM ^2^	*p*-Value ^3^	
	0	30	60	90	120		L	Q
Intake, g/d								
Dry matter ^4^	1279.3	1093.4	977.2	966.9	782.8	32.7	<0.001	0.503
Crude protein ^5^	198.0	169.4	152.4	147.1	119.8	5.56	<0.001	0.592
Ether extract ^6^	42.8	72.3	98.0	128.7	133.3	6.68	<0.001	0.002
aNDFomp ^7^	297.3	245.4	206.4	203.0	187.1	8.18	<0.001	0.005
Non-fibrous carbohydrates ^8^	709.6	576.8	492.4	444.2	352.3	23.0	<0.001	0.173
Total digestible nutrients ^9^	935.6	851.1	860.4	773.3	764.3	26.8	0.004	0.733
Intake, g/kg BW								
Dry matter ^10^	32.1	27.4	27.6	25.0	23.6	0.58	<0.001	0.143
aNDFomp ^11^	7.24	6.20	5.90	5.46	5.07	0.16	<0.001	0.010
Apparent digestibility coefficient, %								
Dry matter	73.5	74.0	74.4	71.5	75.6	1.8	0.782	0.561
Crude protein ^12^	66.2	67.0	71.1	68.6	74.2	2.2	0.016	0.720
Ether extract ^13^	82.1	85.7	88.7	87.6	88.0	1.4	0.005	0.061
aNDFomp ^14^	46.7	48.1	46.9	47.2	57.7	2.9	0.033	0.083
Non-fibrous carbohydrates ^15^	88.0	87.8	86.7	82.9	83.7	1.4	0.005	0.912
Total digestible nutrients ^16^	74.7	78.9	83.1	83.7	91.1	1.8	<0.001	0.713
Performance								
Initial body weight, kg	35.5	35.3	35.3	35.1	33.9	0.45	0.204	0.403
Final body weight, kg ^17^	43.9	45.8	42.6	41.6	36.9	0.70	<0.001	0.122
Average daily gain, g/day ^18^	239.3	211.4	192.0	162.5	93.7	10.9	<0.001	0.105
Feeding efficiency, g gain/Kg DMI ^19^	186.9	190.0	171.9	167.5	119.8	7.22	0.001	0.026

^1^ aNDFomp, Neutral detergent fiber assayed with a heat-stable amylase and expressed exclusive of residual ash and protein; ^2^ SEM, Standard error of the mean; ^3^ L, Linear effect; Q, Quadratic effect; Regression equations: ^4^ DM intake = 1246.97 − 3.6607 × SO, R^2^ = 0.94; ^5^ CP intake = 201.0 − 0.6546 × SO, R^2^ = 0.96; ^6^ EE intake = 42.1077 + 1.3009 × SO − 0.00406 × SO^2^, R^2^ = 0.99; ^7^ aNDFomp intake = 301.4 − 1.917 × SO + 0.008087 × SO^2^, R^2^ = 0.98; ^8^ NFC intake = 685.03 − 2.8187 × SO, R^2^ = 0.96; ^9^ TDN intake = 921.30 − 1.4018 × SO, R^2^ = 0.89; ^10^ DM intake (BW) = 30.878 − 0.0633 × SO, R^2^ = 0.94; ^11^ aNDFomp intake (BW) = 7.015 − 0.0172 × SO, R^2^ = 0.99; ^12^ CP apparent digestibility = 65.97 + 0.06 × SO, R^2^ = 0.73; ^13^ EE apparent digestibility = 83.62 + 0.045 × SO, R^2^ = 0.66; ^14^ aNDFomp apparent digestibility = 45.23 + 0.068 × SO, R^2^ = 0.50; ^15^ NFC apparent digestibility = 88.51 − 0.045 × SO, R^2^ = 0.82; ^16^ TDN apparent digestibility = 74.7870 + 0.1255 × SO, R^2^ = 0.95; ^17^ Final body weight = 44.1409 + 0.04402 × SO − 0.00087 × SO^2^, R^2^ = 0.94; ^18^ Average daily gain = 246.54 − 1.1245 × SO, R^2^ = 0.94; ^19^ Feeding efficiency = 197.97 − 0.5141 × SO, R^2^ = 0.99.

**Table 3:** Ingestive behaviour of feedlot lambs fed diets containing soybean oil.

Item	Soybean Oil Level, g/kg Diet DM					SEM ^1^	*p*-Value	
	0	30	60	90	120		L ^2^	Q ^3^
Times, h/day								
Feeding ^4^	2.5	2.2	2.5	2.9	3.0	0.2	0.004	0.158
Rumination	6.8	6.3	6.4	6.3	5.9	0.3	0.122	0.985
Idling	14.7	15.5	15.1	14.8	15.1	0.4	0.906	0.590
Efficiency, g DM/h								
Feeding ^5^	493.4	488.6	412.4	330.1	295.5	25.8	<0.001	0.502
Rumination ^6^	185.9	174.5	162.6	152.1	140.9	10.3	0.001	0.961
Efficiency, g NDF/h								
Feeding ^7^	110.5	106	92.4	74.5	68.5	5.5	<0.001	0.723
Rumination ^8^	41.9	37.8	36.2	34.1	32.8	1.8	0.001	0.548
Chews								
gDM per bolus ^9^	2.1	2.2	2.3	2.0	1.7	0.1	0.036	0.037
Number of ruminations per bolus ^10^	64.0	73.4	78.1	69.6	70.0	2.6	0.329	0.003
Rumination time per bolus, sec ^11^	40.6	46.0	50.6	44.3	45.2	1.7	0.165	0.003
Number of ruminated boluses ^12^	606.4	492.6	460.3	516.0	476.2	29.5	0.016	0.040
Total chewing times, h/day	9.3	8.5	8.9	9.2	8.9	0.4	0.907	0.590

^1^ S.E.M., Standard error of the mean; ^2^ L, Linear effect; ^3^ Q, Quadratic effect; Regression equations: ^4^ Feeding time = 2.3270 + 0.005 × SO, R^2^ = 0.63; ^5^ Feeding efficiency of DM = 514.83 − 1.8477 × SO, R^2^ = 0.95; ^6^ Rumination efficiency of DM = 185.66 − 0.3747 × SO, R^2^ = 0.99; ^7^ Feeding efficiency of NDF = 113.49 − 0.3849 × SO, R^2^ = 0.96; ^8^ Rumination efficiency of NDF = 40.7070 − 0.0701 × SO, R^2^ = 0.95; ^9^ gDM per bolus = 2.1057 + 0.0076 × SO − 0.00009 × SO^2^, R^2^ = 0.95; ^10^ Number of rumination per bolus = 64.9280 + 0.3242 × SO − 0.00247 × SO^2^, R^2^ = 0.70; ^11^ Rumination time per bolus = 41.0073 + 0.2149 × SO − 0.00158 × SO^2^, R^2^ = 0.66; ^12^ Number of ruminated boluses = 591.38 − 3.0367 × SO + 0.01872 × SO^2^, R^2^ = 0.72.

**Table 4:** Blood metabolites of the protein and energetic profile of feedlot lambs fed diets containing soybean oil.

Item	Soybean Oil Level, g/kg Diet DM					SEM ^1^	*p*-Value	
	0	30	60	90	120		L ^2^	Q ^3^
Total proteins, g/dL ^4^	6.5	6.4	6.7	7.2	7.1	0.1	<0.001	0.760
Albumin (A), g/dL	2.6	2.6	2.6	2.6	2.5	0.1	0.940	0.183
Globulin (G), g/dL ^5^	3.9	3.9	4.1	4.6	4.6	0.1	<0.001	0.443
A:G ratio ^6^	0.66	0.7	0.7	0.6	0.6	0.0	<0.001	0.175
N-ureic, g/dL ^7^	20.3	18.4	15.9	10.5	11.1	0.8	<0.001	0.506
Glucose, g/dL ^8^	83.3	76.2	72.7	69.7	63.1	2.4	<0.001	0.848
Total cholesterol, mg/dL	54.2	59.5	55.1	60.3	54.7	4.8	0.910	0.589
Triglycerides, mg/dL ^9^	46.0	27.4	32.3	29.3	44.5	2.3	0.875	<0.001
Alanine aminotransferase, UI/L	13.7	13.4	15.5	12.3	12.6	1.0	0.295	0.287
Aspartate aminotransferase, UI/L ^10^	86.3	99.8	115.0	214.9	163.2	13.8	<0.001	0.393
Gamma-glutamyltransferase, UI/L	74.8	71.3	66.7	78.1	84.6	4.8	0.091	0.058

^1^ S.E.M., Standard error of the mean; ^2^ L, Linear effect; ^3^ Q, Quadratic effect; Regression equations: ^4^ Total proteins = 6.382 + 0.007 × SO, R^2^ = 0.76; ^5^ Globulin = 3.791 + 0.007 × SO, R^2^ = 0.81; ^6^ A:G ratio = 0.68 − 0.01 × SO, R^2^ = 0.85; ^7^ N-ureic = 20.518 − 0.088 × SO, R^2^ = 0.91; ^8^ Glucose = 82.38 − 0.156 × SO, R^2^ = 0.98; ^9^ Triglycerides = 44.359 − 0.576 × SO + 0.005 × SO^2^, R^2^ = 0.83; ^10^ Aspartate aminotransferase = 81.002 + 0.949 × SO, R^2^ = 0.65.

## Data Availability

Data are contained within the article.
